# Comprehensive analysis to identify pseudogenes/lncRNAs-hsa-miR-200b-3p-COL5A2 network as a prognostic biomarker in gastric cancer

**DOI:** 10.1186/s41065-022-00257-6

**Published:** 2022-11-29

**Authors:** Peiyuan Li, Wenbin Ji, Zhiwang Wei, Xiulan Wang, Gangjie Qiao, Chao Gao, Yifan Wang, Feng Qi

**Affiliations:** Department of General Surgery, Tianjin Medical University General Hospital, No. 154, Anshan Road, Heping District, Tianjin, 300052 China

**Keywords:** COL5A2, Competing endogenous RNA (ceRNA), Bioinformatics, Prognosis, Gastric cancer

## Abstract

**Objective:**

Gastric cancer is one of the most common and deadly types of cancer. The molecular mechanism of gastric cancer progression remains unclear.

**Materials and methods:**

Four hub genes were identified through GEO and TCGA database screening and analysis. Prognostic analysis revealed that COL5A2 was the most likely to affect the prognosis of gastric cancer among the four hub genes. The relationships between COL5A2 and clinical variables and immune cell infiltration were analyzed. Then, COL5A2 was analyzed for single-gene differences and related functional enrichment. Using the starBase database for prediction and analysis, miRNAs and pseudogenes/lncRNAs that might combine with COL5A2 were identified; thus, the ceRNA network was constructed. Finally, the network was verified by Cox analysis and qPCR, and a nomogram was constructed.

**Results:**

First, we found that COL5A2, COL12A1, BGN and THBS2 were highly expressed in gastric cancer. COL5A2 had statistical significance in overall survival (OS), disease-specific survival (DSS), and progression-free interval (PFI) analysis. Immune infiltration analysis suggested that COL5A2 might influence the changes in the tumor immune microenvironment. The StarBase database was used to predict that 3 pseudogenes and 7 lncRNAs might inhibit the hsa-miR-200b-3p-COL5A2 axis in gastric cancer. The pseudogenes/lncRNA-hsa-miR-200b-3p-COL5A2 ceRNA network was identified and verified using Cox regression analysis and PCR. Finally, we constructed a nomogram.

**Conclusions:**

We elucidated the regulatory role of the pseudogenes/lncRNA-hsa-miR-200b-3p-COL5A2 network in gastric cancer progression and constructed a nomogram. These studies may provide effective treatments and potential prognostic biomarkers for gastric cancer.

**Supplementary Information:**

The online version contains supplementary material available at 10.1186/s41065-022-00257-6.

## Introduction

As one of the common gastrointestinal malignancies, gastric cancer (GC) is characterized by a high degree of malignancy, rapid development, strong invasiveness and poor prognosis [1, 2]. The global incidence and the death rate of gastric cancer rank fifth and second, respectively [3]. Due to the lack of specificity of early gastric cancer symptoms, most patients are already diagnosed when they are already in the middle and late stages. Therefore, it is important to prolong the survival time of patients with gastric cancer to find and intervene in the expression of genes that are abnormally expressed in gastric cancer.

Collagen is a major component of the stromal extracellular matrix (ECM), and can be classified as types I-V [4]. Type V collagen (COL5), a relatively small component of the ECM, is a kind of collagen that regulates fiber formation and forms heteromorphic fibers with type I collagen, thereby regulating its diameter during fiber formation [5]. There are three main subtypes of COL5, consisting of three different polypeptide α chains (A1, A2 and A3) [5]. Type V collagen α2 chain (COL5A2) plays important roles in immune system regulation, angiogenesis and tumor metastasis, and participates in the occurrence and development of colorectal cancer, breast cancer and osteosarcoma [6–8]. Studies have shown that when the expression of COL5A2 increases, tumor cells show infinite growth and angiogenesis, and the expression levels of related cytokines such as VEGF and p53 increase [9–11]. Therefore, COL5A2 may be a potential biomarker and therapeutic target for gastric cancer [12].

RNAs consist of coding RNAs (messenger RNAs, mRNAs) and noncoding RNAs (ncRNAs). In recent years, ncRNAs have become the center of human genome research [13]. NcRNAs have an important impact on human health, and the imbalance of ncRNAs will leads to many human diseases, including cancer [14, 15]. There are many types of ncRNAs, including microRNAs (miRNAs), long ncRNAs (lncRNAs), and pseudogenes [16, 17]. In 2011, Salmena et al. put forward the hypothesis of competing endogenous RNA (ceRNA), which is a regulatory mechanism between mRNAs and ncRNAs [18]. The ceRNA mechanism proves that lncRNAs/pseudogenes, miRNAs and mRNAs can crosstalk through competitively binding and sharing miRNAs [19]. An increasing number of ncRNAs have been found to be important tumor promoters or inhibitors [20–23]. In addition, ncRNAs can be used as potential biomarkers for cancer diagnosis and prognosis [24–27].

In this study, we identified COL5A2 through dataset screening and prognostic analysis. Then, the single-gene difference of COL5A2 was analyzed, and functional enrichment and protein-protein interaction (PPI) network analyses were performed. The miRNAs bound to *COL5A2* were analyzed by cytoscope and the TCGA database. The StarBase database was used to screen and predict pseudogenes and lncRNAs bound with miRNAs and then to construct a ceRNA network with pseudogenes/lncRNAs. By establishing a pseudogene/lncRNA-miRNA-mRNA network, we have provided new insights into the progression of gastric cancer and may provide effective therapeutic targets and prognostic biomarkers for gastric cancer.

## Materials and methods

### Microarray data analysis and screening of differentially expressed genes

We searched the widely used Gene Expression Omnibus (GEO) database to compare gene expression in gastric cancer and normal tissues. Gene expression profiles of GSE19826, GSE26899, GSE54129, GSE79973 and GSE103236 were screened from the GEO database. Differentially expressed genes (DEGs) were screened from microarray datasets using the limma package in R software [28]. The cutoff conditions were set to an adjusted *P* value< 0.05 and an absolute value of log-fold change | log_2_FC| > 1. The intersection of datasets was determined by using Venn diagrams. In The Cancer Genome Atlas (TCGA) database, ggplot2 in R software was used to analyze the relationship between COL5A2 and common DEGs in gastric cancer data.

### Functional enrichment analysis of DEGs

The ClusterProfiler package [version 3.14.3] in R software was used for enrichment analysis. The org.Hs.eg.db package [version 3.10.0] in R software was used for ID conversion [29]. The common differentially expressed genes of the five datasets were directly analyzed by GO analysis and KEGG analyses. For the single-gene difference analysis based on COL5A2, the visualization of the GO and KEGG analyses was carried out according to the standard of p.adj < 0.1 & qvalue < 0.2. On the basis of the enrichment analysis, it was preliminarily judged whether the corresponding entry was positive or negative regulation by using the provided log_2_FC of molecules. Then, using the provided log_2_FC of molecules, we calculated the corresponding zscore of each entry and preliminarily judged whether the corresponding entry was positive regulation (zscore +) or negative regulation (zscore -).

The ClusterProfiler package in R software was used for Gene Set Enrichment Analysis (GSEA) [30], and the gene set database was MSigDB Collections. Visualization of GSEA was performed using the ggplot2 package in R software.

### PPI network analysis of DEGs

Protein-protein interaction (PPI) network analysis was performed using the STRING database and Cytoscape. MCODE and cytoHubba were used for PPI network analysis and screening. The first four common genes of the two algorithms were used for subsequent analyses.

### Analysis of the relationship between gene expression and clinical variables

RNAseq data in TPM format from TCGA and GTEx were processed by UCSC XENA (https://xenabrowser.net/datapages/) through the Toil process [31]. The corresponding normal tissue data in STAD (gastric cancer) and GTEx of TCGA were extracted. Four genes were analyzed in unpaired samples from cancer and normal tissues. Using the TCGA (https://portal.gdc.cancer.gov/) STAD (stomach) project level 3 HTSeq-RNAseq FPKM format data, the four genes were matched by tumor tissue and normal tissue samples. The Survival package [version 3.2-10] in R software was used for statistical analysis of survival data. The survminer package [version 0.4.9] in R software was used for data visualization.

The relationships between overall survival (OS) events, disease-specific survival (DSS) events, T stage, N stage, M stage, pathologic stage and COL5A2 expression were analyzed by using the ggplot2 package [3.3.3 version] in R software.

In the baseline data sheet, all levels of TNM stage, pathologic stage, gender, race, histological type, residual tumor, antireflux treatment, H pylori infection, Barrett’s oesophagus, OS event, DSS event, and PFI event met the conditions of theoretical frequency > 5 and total sample size > 40, so the chi-square test was used. Primary therapy outcome and histologic grade level did not meet the conditions of theoretical frequency > 5 or total sample size > 40, so Fisher’s test was used.

### Analysis of the relationship between COL5A2 and immune infiltration

Immune infiltration was analyzed by the GSVA package of R software [32]. The data were derived from gastric cancer data in the TCGA database. The selected correlation analysis method was Spearman’s test. The immune cells were aDCs [activated DCs]; B cells; CD8 T cells; cytotoxic cells; DCs; Eosinophils; iDCs [immature DCs]; macrophages; mast cells; neutrophils; NK CD56bright cells; NK CD56dim cells; NK cells; pDCs [plasmacytoid DCs]; T cells; T helper cells; Tcms [T central memory cells]; Tems [T effector memory cells]; Tfhs [T follicular helper cells]; Tgds [T gamma delta cells]; Th1 cells; Th17 cells; and Th2 cells [33].

### Prediction and analysis of relevant pseudogenes/lncRNAs were performed using the starBase database

The starBase database is a widely used open-source platform for studying non-coding RNA (ncRNA)interactions from CLIP-seq, degradome-seq and RNA–RNA interaction data [34, 35]. In this paper, the starBase database was introduced to analyze the correlation between miRNA and gene or pseudogene expression. Pancancer ≥1 cancer type was set as the screening standard for identifying important lncRNA/pseudogene pairs.

### Cell localization of potential upstream lncRNAs

LNCipedia (https://lncipedia.org/) was used to obtain DElncRNA sequences, and the lncLocator (http://www.csbio.sjtu.edu.cn/bioinf/lncLocator/) database was used to identify the DElncRNA cellular localizations based on their sequences.

### Human gastric cancer tissue

The use of human gastric cancer tissues was approved by the Ethics Committee of Tianjin Medical University General Hospital, and informed consent was obtained from all patients. Gastric cancer tissues and normal tissues from three patients were analyzed.

### RNA isolation and quantitative RT–PCR

Total RNA was purified from gastric cancer tissues and normal tissues using TRIzol reagent (Solarbio, China). CDNA was obtained by reverse transcription of RNA. The expression levels of specific genes were analyzed by real-time PCR (Bio-Rad, USA). The mRNA primers and lncRNA primers were designed by Keyybio (Jinan, China). The miRNA primers were designed by RiboBio (Guangzhou, China).

### Clinical statistical analysis of prognosis, model construction and evaluation

The R software package (version 3.6.3) was used for statistical analysis. The survival package [version 3.2-10] was used to analyze the survival data. The variables included T stage, N stage, M stage, pathologic stage, gender, age, histological type, residual tumor, histologic grade; reflux history; antireflux treatment; *H. pylori* infection, Barrett’s esophagus, COL5A2, AC241952.1, HSPA8P4, PHC1P1, RBMS1P1, AC008040.1, AC016727.1, AC025569.1, AL049796.1, LINC01140, LINC01303, MSC-AS1, OIP5-AS1, RRN3P2, ZEB1-AS1and ZNF652P1. The data were RNA-seq data in level 3 HTSeq-FPKM format from the TCGA (https://portal.gdc.cancer.gov/) STAD (gastric cancer) project. Univariate and multivariate Cox analysis were used to compare the effects of COL5A2 expression and other clinical features on the survival rate. The median was used to determine the critical value. The factors with *P* < 0.1 in univariate cox analysis were included in multivariate Cox analysis.

Based on the Cox regression model and RT–PCR, a nomogram was established to predict the 1-, 3- and 5-year survival rates. Data were processed through R (version 3.6.3) (statistical analysis and visualization), the R package “rms” (version 6.2-0) and the survival package (version 3.2-10). By mapping the prediction probability of the nomogram with the observed events, the calibration curve was graphically evaluated, and the 45° lines represented the best predicted value. The coordination index was used to determine the nomogram discrimination.

## Results

### Acquisition of differentially expressed genes (DEGs) in the GEO database

**Fig. 1:**
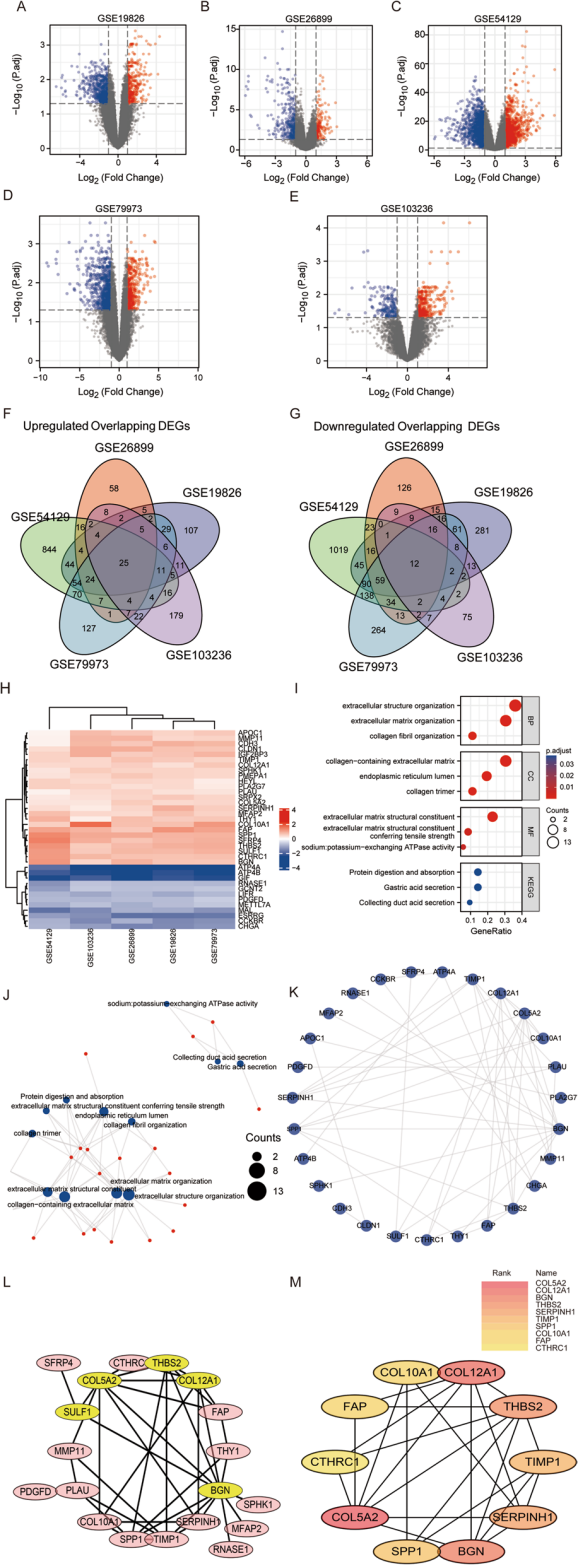
Screening and analysis of DEGs in gastric cancer datasets. Volcano map of differentially expressed genes in the GSE19826 **A**, GSE26899 **B**, GSE54129 **C**, GSE79973 **D**, and GSE103236 **E** datasets. **F** Common upregulated DEGs in the five datasets. **G** Common downregulated DEGs in the five datasets. **H** Heatmaps of 25 upregulated common DEGs and 12 downregulated common DEGs in the five datasets. Each column represents a dataset and each row represents a gene name. **I** GO and KEGG analyses of the common DEGs. **J** GO and KEGG analysis visualization network for common DEGs. **K** PPI network analysis of 37 common DEGs. **L** Hub genes were screened by MCODE. **M** Hub genes were screened by cytoHubba. DEGs, differentially expressed genes; GO, Gene Ontology; KEGG, Kyoto Encyclopedia of Genes and Genomes; PPI, Protein–protein interaction

Each dataset was analyzed with R and screened for DEGs (|log FC| > 1 and adjusted *P* < 0.05). In this study, 239 GC tissues and 67 normal stomach tissues from five GEO datasets were involved (Table 1). A total of 338 upregulated and 643 downregulated genes were filtered from GSE19826; 174 upregulated and 353 downregulated genes from GSE26899; 1134 upregulated and 1449 downregulated genes from GSE54129; 398 upregulated and 728 downregulated genes from GSE79973; and 311 upregulated and 164 downregulated genes from GSE103236. The DEGs in each dataset were presented in a volcano plot, and ggplot2 was used for visualization (Fig. 1A–E). The intersection of upregulated and downregulated genes in five datasets was selected by Venn diagram, and 25 upregulated genes and 12 downregulated genes were screened out (Fig. 1F–G). The 37 selected genes were visually expressed by a heatmap using R (Fig. 1H).

**Table 1:** Details of the five Gene Expression Omnibus gastric cancer data sets

GEO	Tissue	Platform	Normal	Tumor
GSE19826	stomach	GPL570	15	12
GSE26899	stomach	GPL6947	12	96
GSE54129	stomach	GPL570	21	111
GSE79973	stomach	GPL570	10	10
GSE103236	stomach	GPL4133	9	10

### Functional enrichment and analysis of DEGs

Functional enrichment analysis of 37 differentially expressed genes shared by five datasets was performed by GO and KEGG (Fig. 1I). In biological process terms, the DEGs were significantly enriched in extracellular structure organization, extracellular matrix organization, and collagen fibril organization. In cellular component terms, the DEGs were mainly involved in collagen-containing extracellular matrix and endoplasmic reticulum lumen, and collagen trimer. For molecular functions, the DEGs were mainly enriched in extracellular matrix structural constituent, extracellular matrix structural constituent conferring tensile strength, and sodium: potassium-exchanging ATPase activity. The items in which more than 10 genes were enriched were extracellular structure organization, extracellular matrix organization, and collagen-containing extracellular matrix (Fig. 1J).

### Protein–protein interaction (PPI) network analysis

PPI network analysis of 37 differentially expressed genes was performed in the STRING database (Fig. 1K), and hub genes were screened by MCODE (Fig. 1L) and cytoHubba (Fig. 1M) in cytoscope respectively. We selected the first four genes for follow-up analysis: *COL5A2*, *COL12A1*, *BGN*, and *THBS2*.

### Expression and prognostic analysis of DEGs

**Fig. 2:**
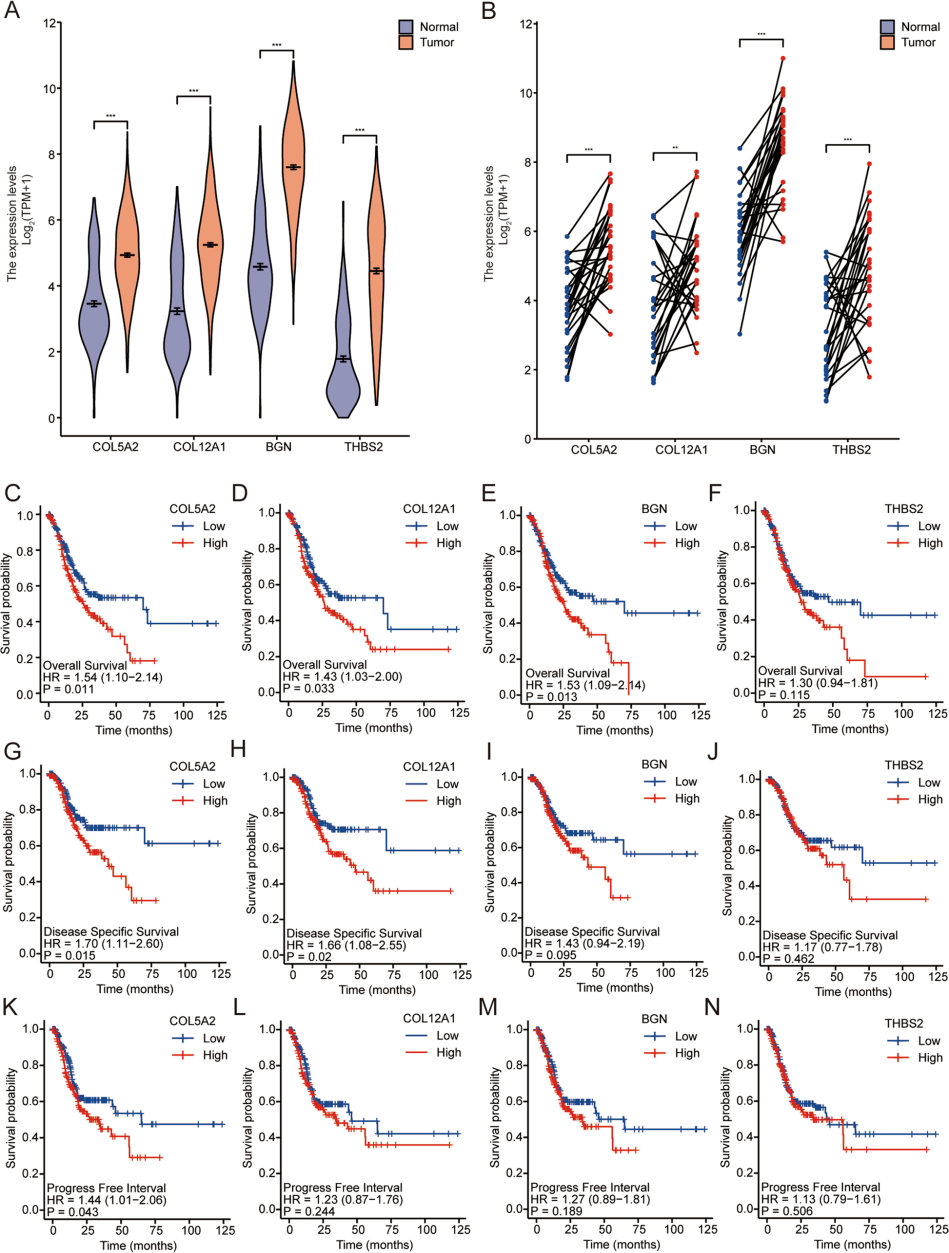
Hub gene expression and prognosis in tissues. **A** Differences in the expression levels of hub genes in unpaired samples. **B** Differences in the expression levels of hub genes in paired samples. Overall survival (OS) of COL5A2 **C**, COL12A1 **D**, BGN **E**, and THBS2 **F**. Disease-specific survival (DSS) of COL5A2 **G**, COL12A1 **H**, BGN **I**, and THBS2 **J**. Progression-free interval (PFI) of COL5A2 **K**, COL12A1 **L**, BGN **M**, and THBS2 **N**

In the TCGA database combined with the GTEX-STAD database, we analyzed the expression profiles of these four genes in 210 normal and 414 cancer samples. We found that the expression levels of these four genes were higher in gastric cancer than in normal tissues (*P* < 0.05) (Fig. 2A). In addition, we compared 407 pairs of gastric cancer samples from the TCGA database and found that 4 genes were highly expressed in cancer tissues (*P* < 0.005) (Fig. 2B).

Subsequently, we divided the four genes into two groups according to high expression and low expression for prognostic analysis. The 10-year overall survival (OS) rate of the group with low expression levels of COL5A2, COL12A1 and BGN was significantly higher than that of the high expression group (*P* < 0.05; Fig. 2C–E). The 10-year OS rate of the THBS2 low expression group was higher than that of the THBS2 high expression group, but the difference was not statistically significant (*P* > 0.05; Fig. 2F). The 10-year disease-special survival (DSS) rate of the group with low expression levels of COL5A2 and COL12A1 was significantly higher than that of the high expression group (P < 0.05; Fig. 2G, H), and there was no statistical significance between THBS2 and BGN expression levels (P > 0.05; Fig. 2I, J). The 10-year progression-free interval (PFI) rate of the group with low expression of COL5A2 was significantly higher than that of the high expression group (*P* < 0.05; Fig. 2K), and the expression levels of THBS2, COL12A1 and BGN were not significantly different from the PFI (*P* > 0.05; Fig. 2L–N).

Because there were significant relationships between the expression of COL5A2 and OS, DSS, and PFI, we carried out a follow-up single-gene analysis of COL5A2.

### Relationship between COL5A2 expression and clinicopathologic variables

**Fig. 3:**
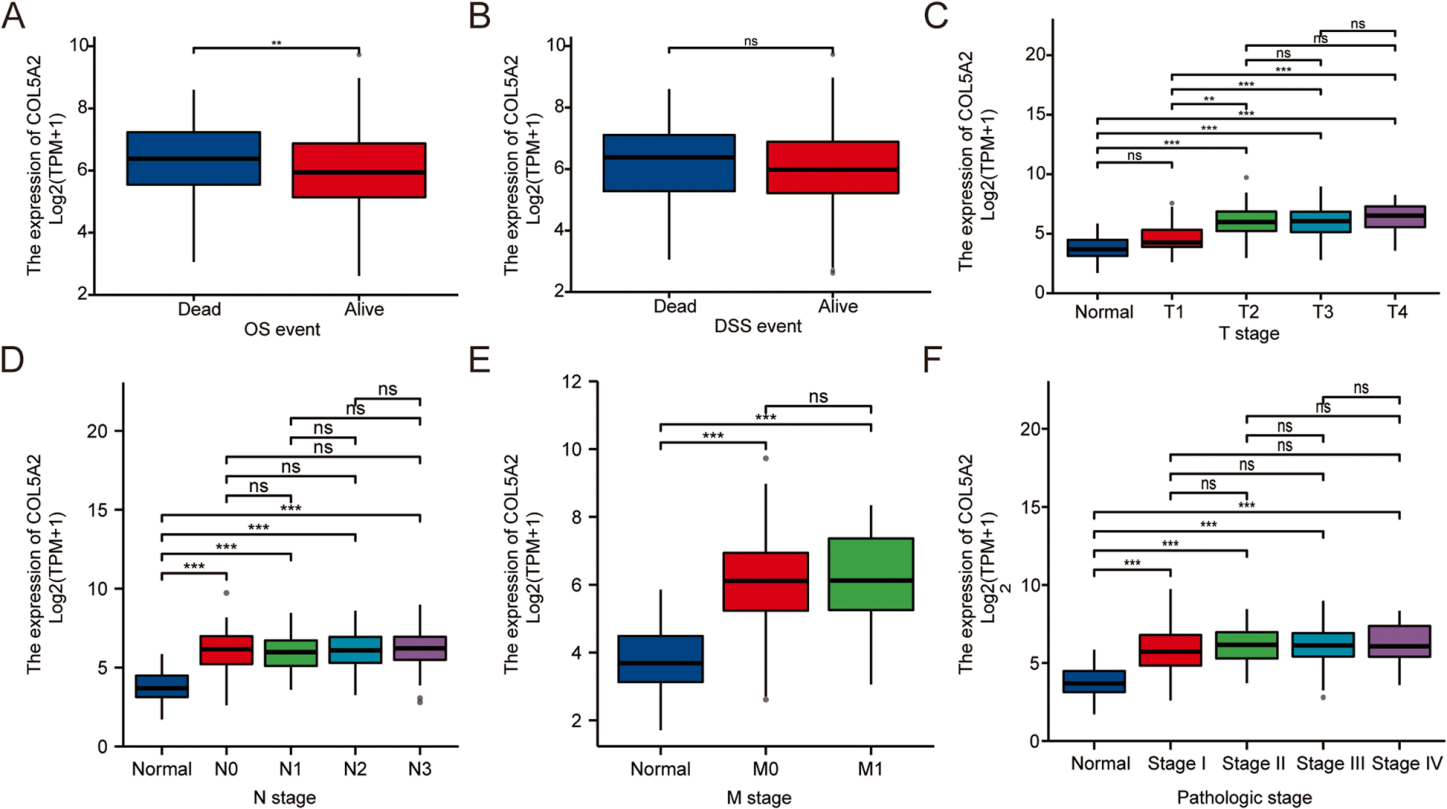
Correlations between COL5A2 expression and clinical data, including **A** overall survival (OS), **B** disease-specific survival (DSS), **C** T stage, **D** N stage, **E** M stage, and **F** pathologic stage

A total of 407 patients with GC were collected by TCGA and divided into two groups according to the expression level of COL5A2 to explore the correlations between COL5A2 expression and clinical indices (Table 2). In the relationship between COL5A2 and clinical data, OS events showed that COL5A2 was different and statistically significant (P < 0.05; Fig. 3A), while DSS events had no statistical significance (P > 0.05; Fig. 3B). In T stage, the difference between the normal and T1 groups was statistically significant compared with the T2, T3 and T4 groups (Fig. 3C). Regarding N stage, M stage and pathologic stage, the differences between the normal group and the other groups were statistically significant (Fig. 3D–F).

**Table 2:** Baseline data sheet

Characteristic	Low expression of COL5A2	High expression of COL5A2	p
n	187	188	
T stage, n (%)			0.009
T1	16 (4.4%)	3 (0.8%)	
T2	44 (12%)	36 (9.8%)	
T3	84 (22.9%)	84 (22.9%)	
T4	43 (11.7%)	57 (15.5%)	
N stage, n (%)			0.772
N0	54 (15.1%)	57 (16%)	
N1	53 (14.8%)	44 (12.3%)	
N2	38 (10.6%)	37 (10.4%)	
N3	35 (9.8%)	39 (10.9%)	
M stage, n (%)			0.965
M0	167 (47%)	163 (45.9%)	
M1	12 (3.4%)	13 (3.7%)	
Pathologic stage, n (%)			0.342
Stage I	33 (9.4%)	20 (5.7%)	
Stage II	53 (15.1%)	58 (16.5%)	
Stage III	74 (21%)	76 (21.6%)	
Stage IV	19 (5.4%)	19 (5.4%)	
Primary therapy outcome, n (%)			0.860
PD	35 (11%)	30 (9.5%)	
SD	7 (2.2%)	10 (3.2%)	
PR	2 (0.6%)	2 (0.6%)	
CR	118 (37.2%)	113 (35.6%)	
Gender, n (%)			0.313
Female	72 (19.2%)	62 (16.5%)	
Male	115 (30.7%)	126 (33.6%)	
Race, n (%)			0.083
Asian	37 (11.5%)	37 (11.5%)	
Black or African American	9 (2.8%)	2 (0.6%)	
White	113 (35%)	125 (38.7%)	
Age, n (%)			0.422
< =65	87 (23.5%)	77 (20.8%)	
> 65	100 (27%)	107 (28.8%)	
Histological type, n (%)			0.056
Diffuse Type	27 (7.2%)	36 (9.6%)	
Mucinous Type	6 (1.6%)	13 (3.5%)	
Not Otherwise Specified	100 (26.7%)	107 (28.6%)	
Papillary Type	3 (0.8%)	2 (0.5%)	
Signet Ring Type	6 (1.6%)	5 (1.3%)	
Tubular Type	45 (12%)	24 (6.4%)	
Residual tumor, n (%)			0.886
R0	157 (47.7%)	141 (42.9%)	
R1	7 (2.1%)	8 (2.4%)	
R2	8 (2.4%)	8 (2.4%)	
Histologic grade, n (%)			0.152
G1	5 (1.4%)	5 (1.4%)	
G2	77 (21%)	60 (16.4%)	
G3	100 (27.3%)	119 (32.5%)	
Reflux history, n (%)			0.286
No	93 (43.5%)	82 (38.3%)	
Yes	25 (11.7%)	14 (6.5%)	
Antireflux treatment, n (%)			0.321
No	69 (38.5%)	73 (40.8%)	
Yes	22 (12.3%)	15 (8.4%)	
H pylori infection, n (%)			0.959
No	86 (52.8%)	59 (36.2%)	
Yes	10 (6.1%)	8 (4.9%)	
Barretts esophagus, n (%)			0.790
No	115 (55.3%)	78 (37.5%)	
Yes	10 (4.8%)	5 (2.4%)	
OS event, n (%)			0.022
Alive	125 (33.3%)	103 (27.5%)	
Dead	62 (16.5%)	85 (22.7%)	
DSS event, n (%)			0.038
Alive	142 (40.1%)	121 (34.2%)	
Dead	37 (10.5%)	54 (15.3%)	
PFI event, n (%)			0.164
Alive	132 (35.2%)	119 (31.7%)	
Dead	55 (14.7%)	69 (18.4%)

### Correlations between COL5A2 and immune cells

**Fig. 4:**
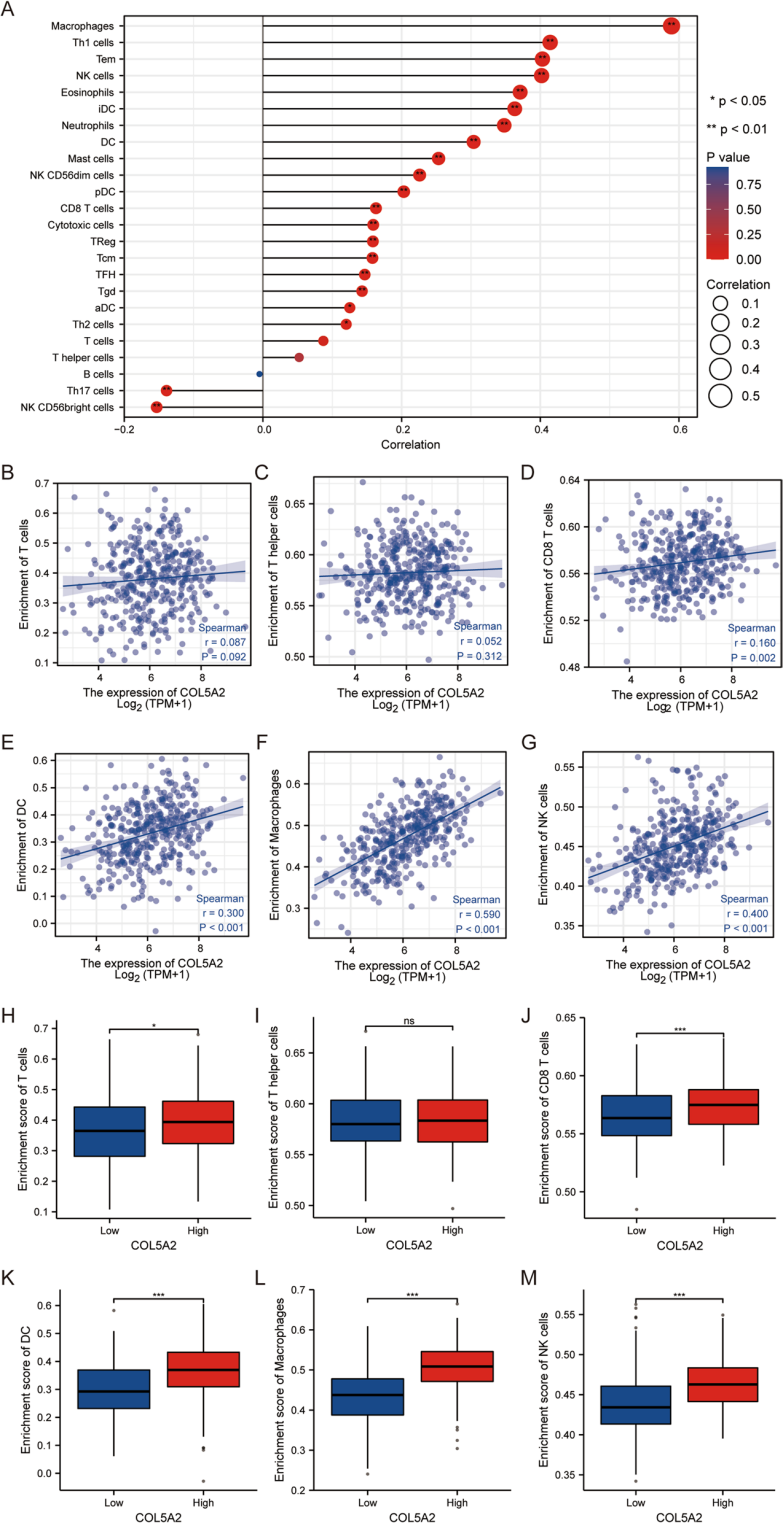
Relationships between COL5A2 expression and immune cell infiltration in gastric cancer. **A** Correlations between COL5A2 and 24 kinds of immune cells. Correlation between COL5A2 expression and related immune cells, including **B** T cells, **C** T helper cells, **D** CD8^+^ T cells, **E** DCs, **F** macrophages, and **G** NK cells. The enrichment degrees of immune cells were compared between the high and low COL5A2 expression groups. **H** T cells, **I** T helper cells, **J** CD8^+^ T cells, **K** DCs, **L** macrophages, **M** NK cells

The correlations between COL5A2 and infiltrating immune cells were calculated by ssGSEA. We found that the expression level of COL5A2 was positively correlated with macrophages, Th1 cells, Tems [T effector memory cells], NK cells, eosinophils, iDCs [IMMATURE DCs], neutrophils, DCs, mast cells, NK CD56dim cells, pDCs [plasmacytoid DCs], CD8 T cells, cytotoxic cells, Tregs, Tcms [T central memory cells], Tfhs [T follicular helper cells], Tgds [T gamma delta cells], aDCs [activated DCs], and Th2 cells, and negatively correlated with Th17 cells and NK CD56bright cells (Fig. 4A).

Through the correlation analysis of COL5A2 and immune cells, we found that COL5A2 was positively correlated with T cells, CD8 T cells, DCs, macrophages, and NK cells (Fig. 4B–G). Similarly, macrophages and NK cells were divided into two groups according to the expression level of COL5A2. T cells, CD8 T cells, DCs, macrophages and NK cells showed higher enrichment scores in the group with high expression levels of COL5A2 (Fig. 4H–M).

### Functional enrichment and analysis of COL5A2 in GC

**Fig. 5:**
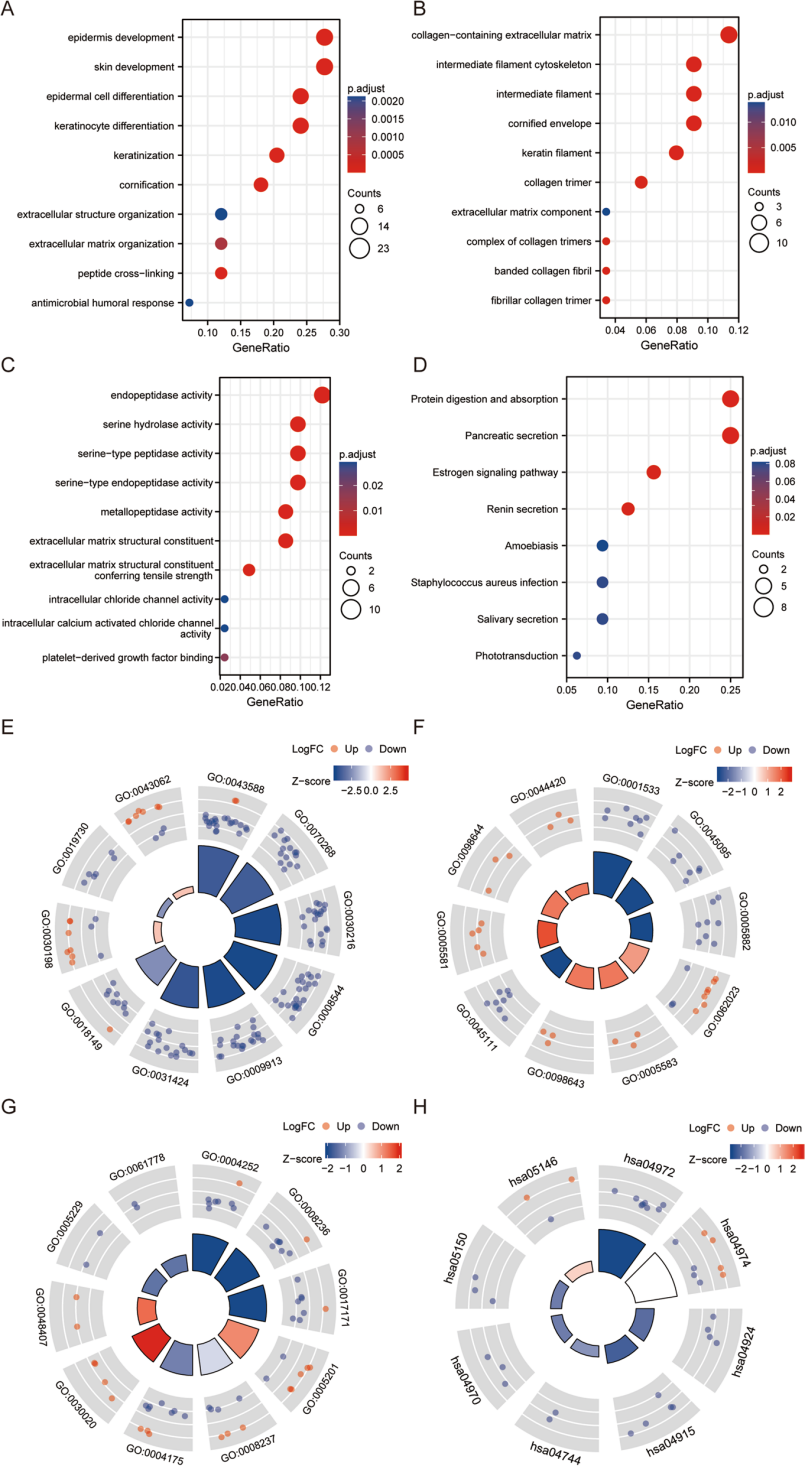
GO and KEGG analyses of the DEGs. **A** Biological process. **B** Cell component. **C** Molecular function. **D** KEGG pathway. **E** Biological process (log_2_FC). **F** Cell component (log_2_FC). **G** Molecular function (log_2_FC). **H** KEGG pathway (log_2_FC)

The two groups were divided into high and low expression of COL5A2, and the expression profiles of differentially expressed genes in the two groups were analyzed. According to |log_2_FC| > 2, and p.adj < 0.05, 116 differentially expressed genes were screened. GO and KEGG enrichment analyses were performed for 116 differentially expressed genes (Table 3). In biological processes, COL5A2 was closely related to epidermal development, skin development, and epidermal cell differentiation (Fig. 5A). In cell components, there were certain correlations between COL5A2 and collagen-containing extracellular matrix, intermediate filament cytoskeleton, and intermediate filament (Fig. 5B). In molecular functions, COL5A2 was associated with endopeptidase activity, serine hydrolase activity, and serine-type peptidase activity (Fig. 5C). In the KEGG pathway analysis, COL5A2 was related to protein digestion and absorption, pancreatic secretion, and the estrogen signaling pathway (Fig. 5D). After that, we conducted functional enrichment and log_2_FC analysis on the differentially expressed genes to preliminarily determine whether the corresponding items were positively or negatively regulated (Fig. 5E–H).

**Table 3:** *COL5A2* related differentially expressed genes analyzed by GO and KEGG

ONTOLOGY	ID	Description	GeneRatio	BgRatio	pvalue	p.adjust	qvalue
BP	GO:0043588	skin development	23/83	419/18670	3.47e-19	4.23e-16	4.03e-16
BP	GO:0070268	cornification	15/83	112/18670	1.54e-18	9.40e-16	8.95e-16
BP	GO:0030216	keratinocyte differentiation	20/83	305/18670	3.18e-18	1.02e-15	9.71e-16
BP	GO:0008544	epidermis development	23/83	464/18670	3.35e-18	1.02e-15	9.71e-16
BP	GO:0009913	epidermal cell differentiation	20/83	358/18670	7.25e-17	1.77e-14	1.68e-14
CC	GO:0001533	cornified envelope	8/88	65/19717	4.67e-10	4.67e-08	4.23e-08
CC	GO:0045095	keratin filament	7/88	95/19717	2.23e-07	1.11e-05	1.01e-05
CC	GO:0005882	intermediate filament	8/88	214/19717	5.16e-06	1.72e-04	1.56e-04
CC	GO:0062023	collagen-containing extracellular matrix	10/88	406/19717	1.33e-05	2.30e-04	2.08e-04
CC	GO:0005583	fibrillar collagen trimer	3/88	11/19717	1.38e-05	2.30e-04	2.08e-04
MF	GO:0004252	serine-type endopeptidase activity	8/82	160/17697	7.57e-07	9.09e-05	6.62e-05
MF	GO:0008236	serine-type peptidase activity	8/82	182/17697	2.00e-06	9.40e-05	6.85e-05
MF	GO:0017171	serine hydrolase activity	8/82	186/17697	2.35e-06	9.40e-05	6.85e-05
MF	GO:0005201	extracellular matrix structural constituent	7/82	163/17697	1.05e-05	3.16e-04	2.30e-04
MF	GO:0008237	metallopeptidase activity	7/82	181/17697	2.08e-05	4.98e-04	3.63e-04
KEGG	hsa04972	Pancreatic secretion	8/32	102/8076	4.02e-09	1.91e-07	1.62e-07
KEGG	hsa04974	Protein digestion and absorption	8/32	103/8076	4.34e-09	1.91e-07	1.62e-07
KEGG	hsa04924	Renin secretion	4/32	69/8076	1.47e-04	0.004	0.004
KEGG	hsa04915	Estrogen signaling pathway	5/32	138/8076	1.88e-04	0.004	0.004
KEGG	hsa04744	Phototransduction	2/32	28/8076	0.005	0.079	0.067

**Fig. 6:**
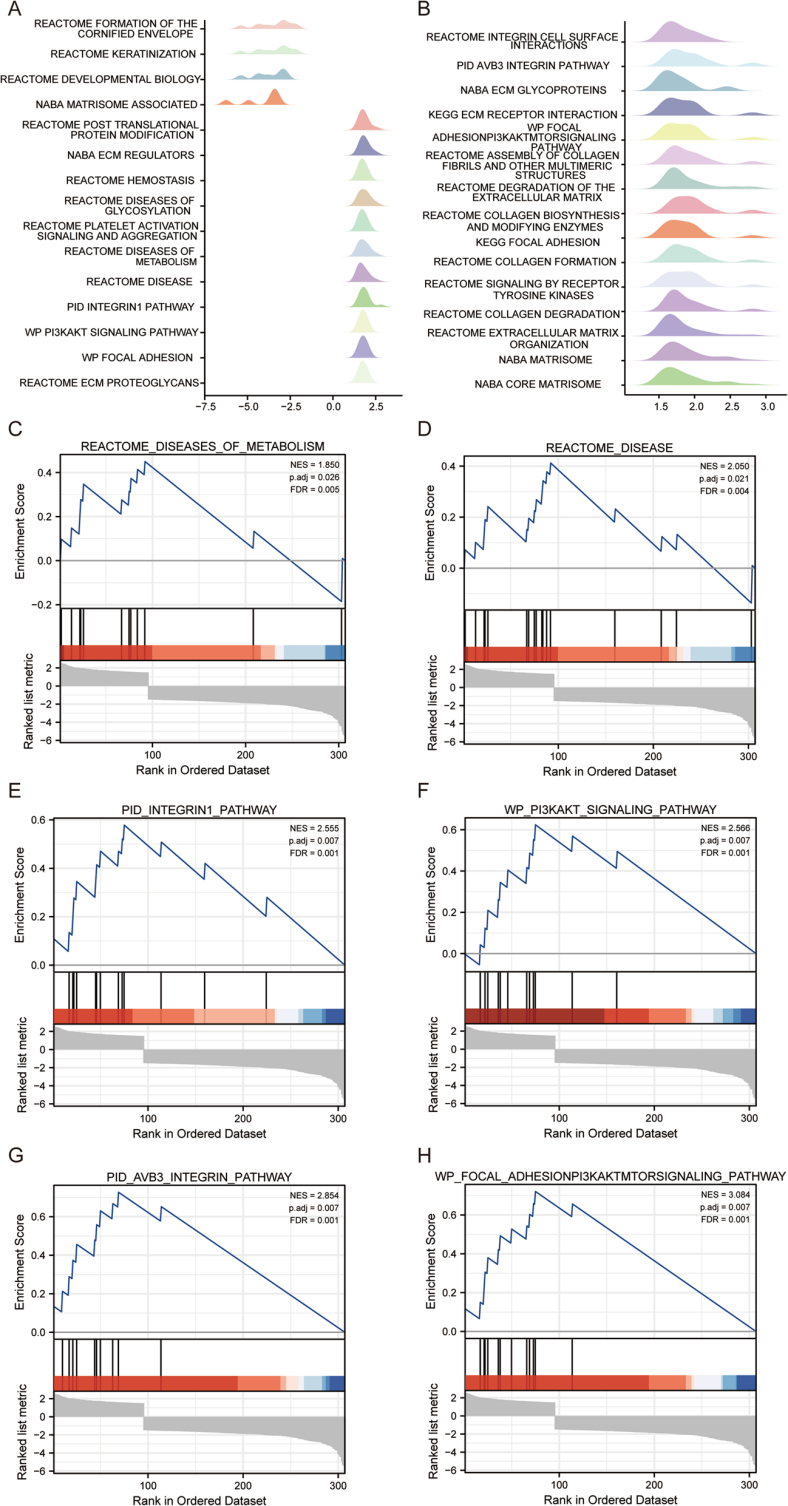
Enrichment plots from the GSEA. **A, B** Differential gene enrichment for all significantly enriched phenotypes. **C-H** Enrichment plots from the GSEA. GC associated with COL5A2 has a variety of pathways and biological processes. Several pathways and biological processes significantly enriched in COL5A2-related GC were identified. NES, normalized enrichment score; p.adj, adjusted P–value; FDR, false discovery rate. GC, gastric cancer; GSEA, Gene Set Enrichment Analysis

According to |log_2_FC| > 1.5, and p.adj < 0.05, 370 differentially expressed genes were screened. GSEA analysis was performed on 370 different genes, and 30 related items were identified (Fig. 6A–B). Based on the specific analysis of related phenotypes and pathways, it was found that the genes related to COL5A2 were positively correlated with REACTOME_DISEASES_OF_METABOLISM, REACTOME_DISEASE,

PID_INTEGRIN1_PATHWAY, WP_PI3KAKT_SIGNALING_PATHWAY,

PID_AVB3_INTEGRIN_PATHWAY,

and WP_FOCAL_ADHESIONPI3KAKTMTORSIGNALING_PATHWAY (Fig. 6C–H).

### Construction of the hsa-miR-200b-3p-COL5A2 axis associated with gastric cancer progression

**Fig. 7:**
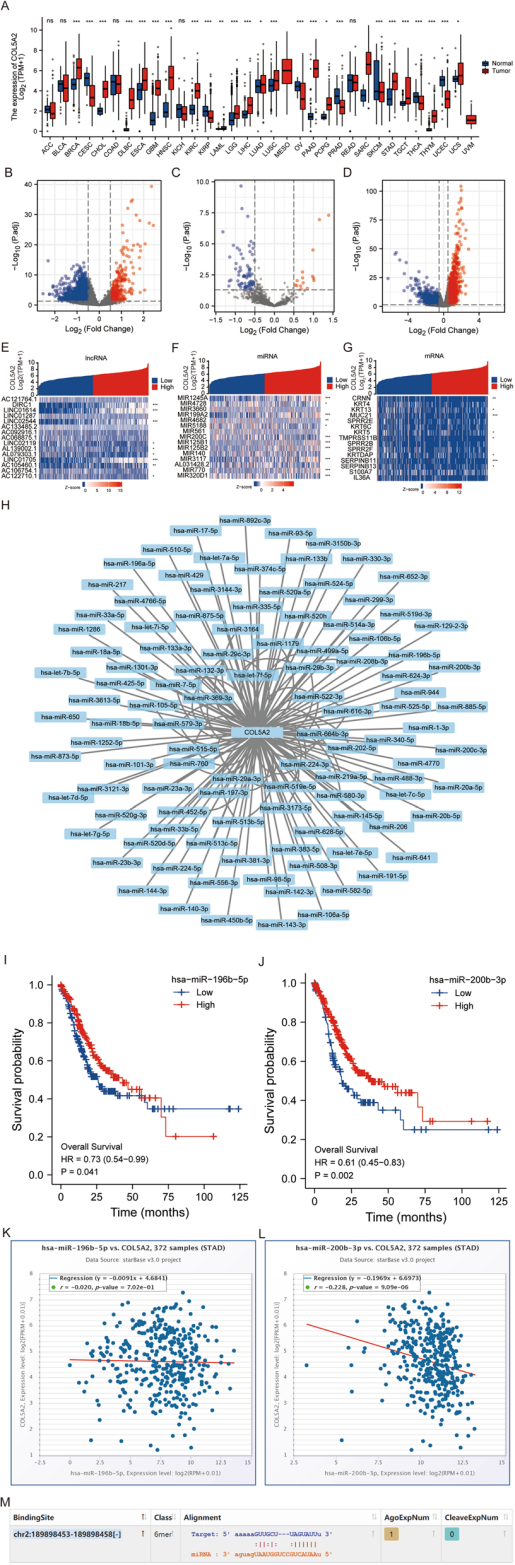
Expression of COL5A2 in different cancers and its relationship with related genes. **A** COL5A2 expression in different cancers in the UCSC XENA database compared with normal tissues. **B** Volcano plot of differentially expressed lncRNAs. **C** Volcano plot of differentially expressed miRNAs. **D** Volcano plot of differentially expressed mRNAs. **E** Correlations between the first 15 significantly different lncRNAs and COL5A2 expression trends. **F** Correlations between the first 15 significantly different miRNAs and COL5A2 expression trends. **G** Correlations between the first 15 significantly differentially expressed mRNAs and COL5A2 expression trend. **H** PPI network diagram of COL5A2 and predicted miRNAs. **I** Relationship between hsa-miR-196b-5p expression and prognosis. **J** Relationship between hsa-miR-200b-3p expression and prognosis. **K** Expression relationship between COL5A2 and hsa-miR-196b-5p. **L** Expression relationship between COL5A2 and hsa-miR-200b-3p. **M** Predicted binding sites of COL5A2 to hsa-miR-200b-3p

The pancancer analysis of COL5A2 was carried out by using the UCSC XENA database. COL5A2 was significantly expressed in breast invasive carcinoma (BRCA), cervical squamous cell carcinoma and endocervical adenocarcinoma (CESC), cholangiocarcinoma (CHOL), lymphoid neoplasm diffuse large B-cell lymphoma (DLBC), esophageal carcinoma (ESCA), glioblastoma multiforme (GBM), head and neck squamous cell carcinoma (HNSC), kidney renal clear cell carcinoma (KIRC), kidney renal papillary cell carcinoma (KIRP), brain lower grade glioma (LGG), liver hepatocellular carcinoma (LIHC), lung squamous cell carcinoma (LUSC), ovarian serous cystadenocarcinoma (OV), pancreatic adenocarcinoma (PAAD), prostate adenocarcinoma (PRAD), skin cutaneous melanoma (SKCM), stomach adenocarcinoma (STAD), testicular germ cell tumors (TGCT), thyroid carcinoma (THCA), thymoma (THYM), and uterine corpus endometrial carcinoma (UCEC). (*P* < 0.01) (Fig. 7A).

Single-gene difference analysis of COL5A2 was carried out, and the differential lncRNAs, miRNAs, and mRNAs were screened according to the standard of | log_2_FC | > 0.05, p adj < 0.05. The related differentially expressed genes were visualized by volcano plots (Fig. 7B–D). Among the different lncRNAs, miRNAs and mRNAs, the first 15 genes were selected to be coexpressed with COL5A2 by sequencing | log_2_FC | from high to low (Fig. 7E–G). The starBase database was used to predict miRNA binding to *COL5A2*. Cytoscape was used to analyze the PPI network (Fig. 7H). Survival analysis of differential miRNAs was performed using the data from the TCGA database of GC. We looked for miRNAs that were positively correlated with prognosis. Only the prognoses of hsa-miR-196b-5p and hsa-miR-200b-3p were significantly different (Fig. 7I, J). Then, we analyzed the relationships between COL5A2 and hsa-miR-196b-5p, and hsa-miR-200b-3p using starBase database. We found that both hsa-miR-196b-5p and hsa-miR-200b-3p were negatively correlated with COL5A2; however, only the negative correlation between hsa-miR-200b-3p and COL5A2 was statistically significant (Fig. 7K, L). Therefore, we hypothesized that hsa-miR-200b-3p might be an miRNA binding to COL5A2. Using the starBase database, we identified a binding site between hsa-miR-200b-3p and COL5A2 (Fig. 7M).

### Potential pseudogenes and lncRNAs upstream of hsa-miR-200b-3p

**Fig. 8:**
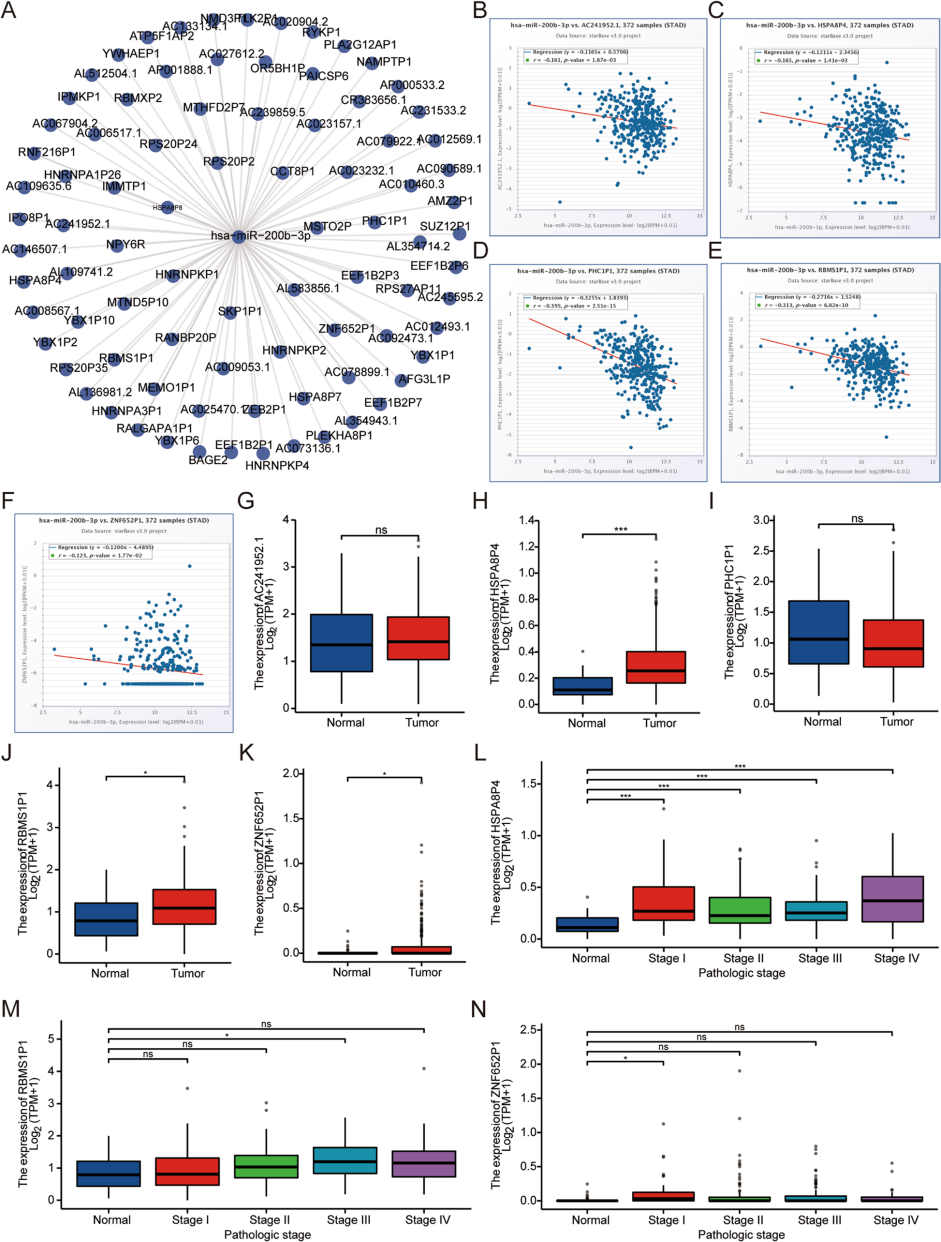
Prediction of potential pseudogenes upstream of hsa-miR-200b-3p in gastric cancer. **A** Pseudogene–hsa-miR-200b-3p network constructed by cytoscope. The relationships between the expression of hsa-miR-200b-3p and pseudogenes, including AC241952.1 **B**, HSPA8P4 **C**, PHC1P1 **D**, RBMS1P1 **E**, and ZNF652P1 **F**. **G–K** Expression of pseudogenes in tumor and normal tissues. **L–N** Expression levels of pseudogenes in normal and different pathological stages. “*” represents “*P* value < 0.05”. “***” represents “*P* value < 0.001”. The Y-axis indicates the relative expression value, log_2_(TPM + 1). TPM = transcripts per million

Pseudogenes and lncRNAs are two important subtypes of noncoding RNAs (ncRNAs), which may act as ceRNAs by competing for shared miRNAs and interacting with mRNAs. Pseudogene binding to hsa-miR-200b-3p was predicted using the starBase database (Fig. 8A). Pseudogenes whose expression was negatively correlated with hsa-miR-200b-3p were analyzed by the starBase database. We found that the expression of hsa-miR-200b-3p was significantly negatively correlated with those of AC241952.1, HSPA8P4, PHC1P1, RBMS1P1, and ZNF652P1 (*P* < 0.05) (Fig. 8B–F). The TCGA database was used to analyze the relationships between the expression levels of pseudogenes in tumor and normal tissues. Except for AC241952.1 and PHC1P1, the expression levels of other predicted pseudogenes were significantly higher in tumor tissues than in normal tissues (P < 0.05) (Fig. 8G–K). After that, we further analyzed HSPA8P4, RBMS1P1 and ZNF652P1. By analyzing the expression profiles of pseudogenes in normal and different stages in the TCGA database, we found that the expression levels of HSPA8P4, RBMS1P1 and ZNF652P1 in normal and pathological stages were different to different degrees. (Fig. 8L–N).

**Fig. 9:**
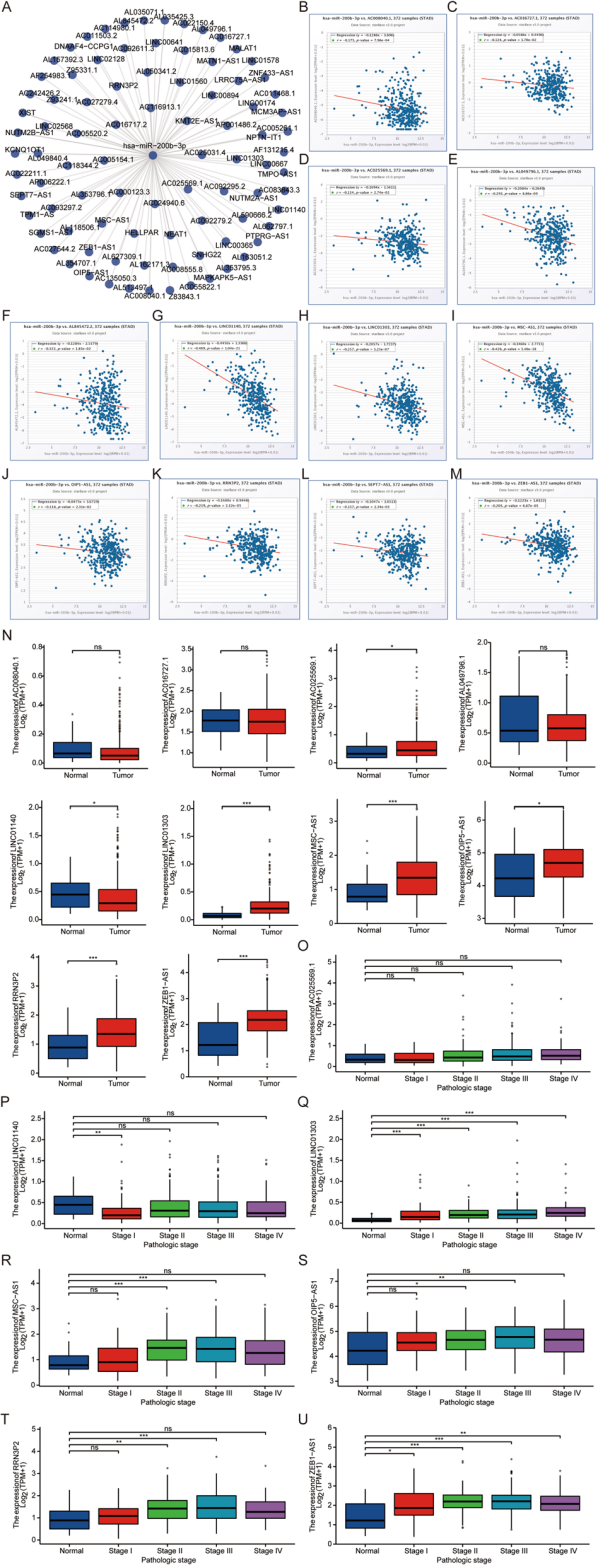
Screening potential lncRNAs upstream of hsa-miR-200b-3p in gastric cancer. **A** The potential lncRNAs of hsa-miR-200b-3p predicted by the starBase database. The relationships between the expression of hsa-miR-200b-3p and lncRNAs, including AC008040.1 **B**, AC016727.1 **C**, AC025569.1 **D**, AL049796.1 **E**, AL845472.2 **F**, LINC01140 **G**, LINC01303 **H**, MSC-AS1 **I**, OIP5-AS1 **J**, RRN3P2 **K**, SEPT7-AS1 **L**, and ZEB1-AS1 **M**. **N** Expression of lncRNAs in tumor and normal tissues. **O–U** Expression levels of lncRNAs in normal and different pathological stages in gastric cancer. “*” represents “*P* value < 0.05”. “**” represents “*P* value < 0.01”. “***” represents “*P* value < 0.001”

Subsequently, lncRNAs associated with hsa-miR-200b-3p were predicted. In the starBase database, the restriction condition was 1 cancer type, and 101 predictive lncRNAs were identified (Fig. 9A). We looked for lncRNAs negatively correlated with hsa-miR-200b-3p expression. With P < 0.05 as the standard for screening, the results are shown in Fig. 9B–M. Except for AC008040.1, AC016727.1 and AL049796.1, the expression levels of other predicted lncRNAs were significantly higher in tumor tissues than in normal tissues (P < 0.05) (Fig. 9N). We then performed a further analysis of the genes expressed at significantly different levels in normal and cancer tissues. By analyzing the expression of lncRNAs in the TCGA database in normal and different stages, we found that the lncRNAs in other normal and pathological stages were expressed to different degrees except for AC025569.1 (Fig. 9O–U).

**Fig. 10:**
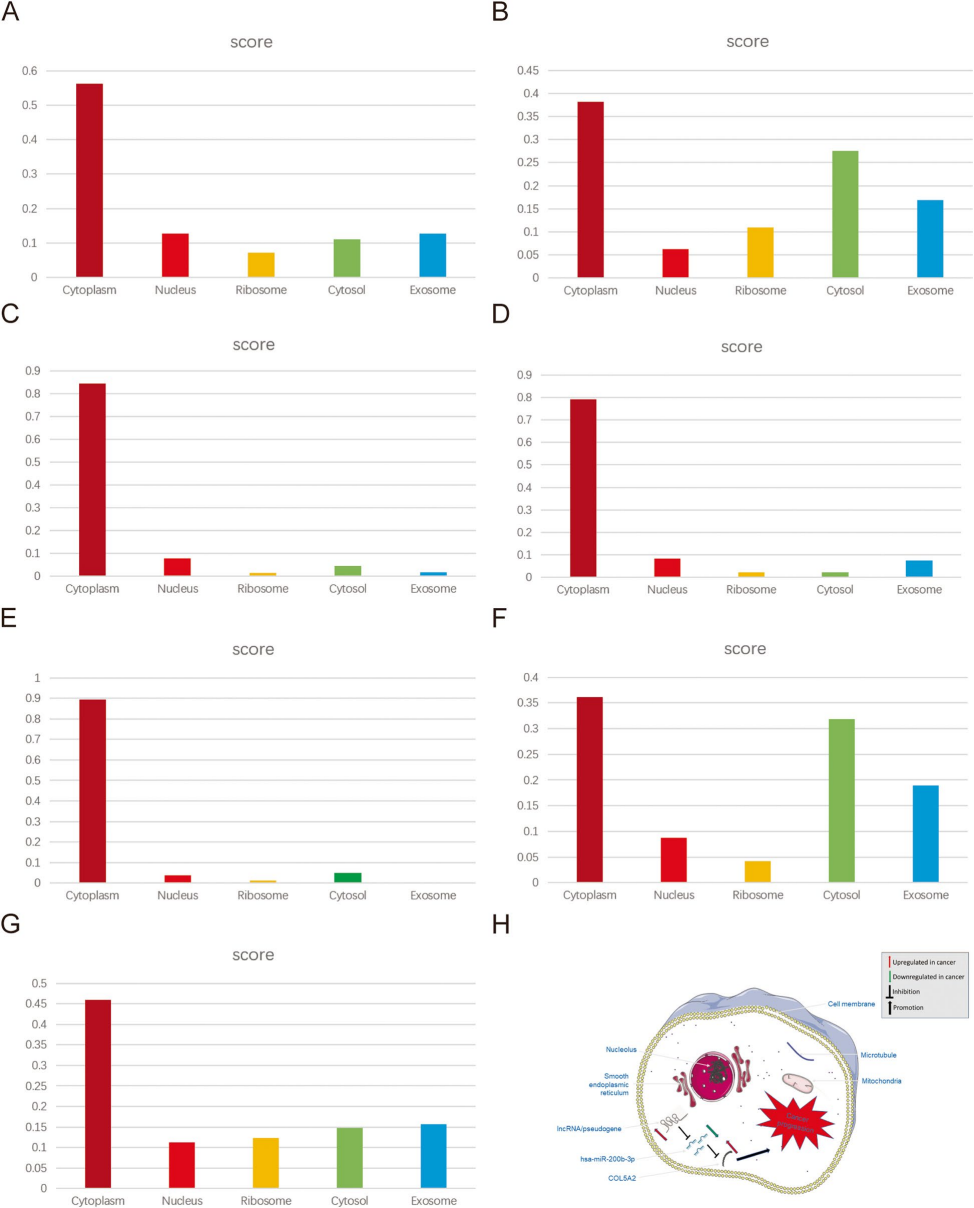
Cell localization of potential upstream lncRNAs. Cell localization prediction performed for lncRNAs, including AC025569.1 **A**, LINC01140 **B**, LINC01303 **C**, MSC-AS1 **D**, OIP5-AS1 **E**, RRN3P2 **F**, and ZEB1-AS1 **G**. **H** Model of the pseudogene/lncRNA-hsa-miR-200b-3p-COL5A2 network and its expression and potential roles in gastric cancer progression

The pseudogenes/lncRNAs were compared with the differential genes in the single-gene analysis of COL5A2, and it was found that these pseudogenes/lncRNAs were all present in the differential genes identified in the single-gene analysis. In addition, considering that the cell localization of lncRNAs determines the underlying mechanism, the LNCipedia database and the lncLocator database were employed for cell localization analysis of lncRNAs with different expression levels in tumor tissues and normal tissues. We found that AC025569.1, LINC01140, LINC01303, MSC-AS1, OIP5-AS1, RRN3P2, and ZEB1-AS1 were mainly distributed in the cytoplasm (Fig. 10A–G). These data suggest that these lncRNAs may act as ceRNAs to influence COL5A2 expression by sponging hsa-miR-200b-3p.

In summary, the downregulation of hsa-miR-200b-3p mediated by overexpressed lncRNAs/pseudogenes leads to an increase in COL5A2 expression, which leads to the progression of gastric cancer (Fig. 10H).

### Establishment of a nomogram of *COL5A2* in gastric cancer

Univariate Cox regression analysis was performed with *P* < 0.1 as the standard. Univariate Cox regression analysis of clinical indicators revealed that T stage, N stage, M stage, pathologic stage, age, histological type, residual tumor, HSPA8P4, PHC1P1, RBMS1P1, LINC01303, and MSC-AS1 were meaningful, and the expression level of COL5A2 was also significant (Table S1). After that, we conducted multivariate Cox regression analysis for the above significant factors. Multivariate Cox regression was used to screen out independent adverse prognostic factors, and we found that N stage, age, histological type, and residual tumor were still meaningful (Table S1).

**Fig. 11:**
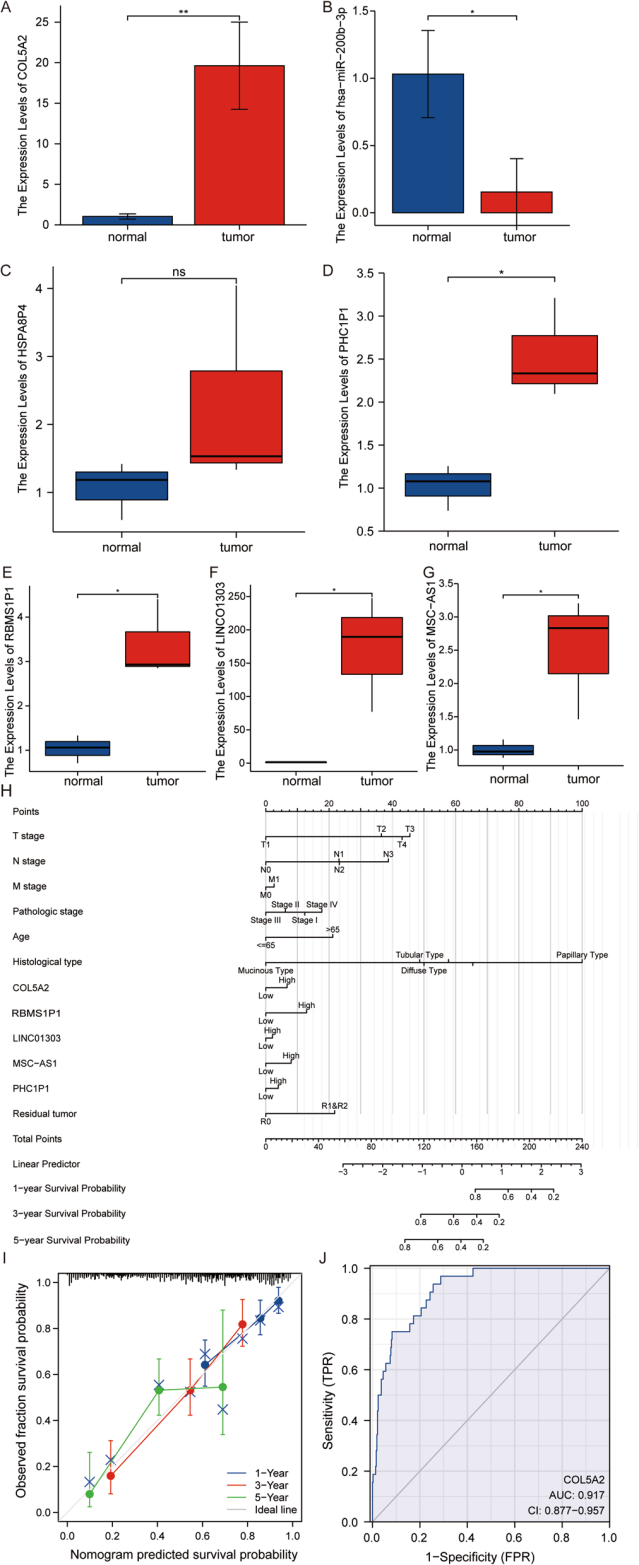
RT–PCR analysis and establishment of a prognosis prediction map for patients with gastric cancer. RNA expression in normal and gastric cancer tissues, including COL5A2 **A**, hsa-miR-200b-3p **B**, HSPA8P4 **C**, PHC1P1 **D**, RBMS1P1 **E**, LINCO1303 **F**, and MSC-AS1 **G**. **H** The 1-, 3- and 5-year predictive survival rates for patients with gastric cancer in the nomogram. **I** Calibration plots comparing predicted and actual OS probabilities at 1-, 3- and 5-year follow-ups. OS, overall survival. **J** A ROC curve established to test the value of COL5A2 to identify GC tissues

Subsequently, we validated the significant RNAs by RT-PCR in gastric cancer tissues and normal tissues. We found that all RNAs were significantly different except HSPA8P4, which was not statistically significant. This result was in line with the network regulatory relationship of ceRNAs (Fig. 11A-G).

We constructed a nomogram using the meaningful factors identified in the univariate Cox regression model (Fig. 11H). The total number of points assigned to each variable was adjusted to 1-100. The integral of each variable was summed and recorded as a total score. The probabilities of survival of patients with gastric cancer at 1, 3 and 5 years were determined by drawing vertical lines directly down from the total point axis to the result axis. The prediction efficiency of the model was analyzed, and the results showed that the C index of the model was 0.737 (0.712-0.762), indicating that the prediction efficiency of the model was medium. Then, we used the calibration diagram for our prognosis analysis. The 1-, 3-, 5-year prognosis and survival curves were basically consistent with the ideal line (Fig. 11I), indicating that this nomogram served as a better model for predicting the short- or long-term survival of patients with gastric cancer. Receiver operating characteristics (ROC) were used to analyze the role of COL5A2 in differentiated carcinoma tissues and normal tissues of GC. The area under the curve of COL5A2 was 0.917, which indicates that COL5A2 has a certain accuracy in predicting tumor and normal outcomes (Fig. 11J).

## Discussion

Because of its invasiveness and recurrence, the treatment of gastric cancer is still a great challenge. The early symptoms of gastric cancer are not significant, so it is necessary to explore the pathogenesis and biomarkers of gastric cancer. Bioinformatics analysis has been widely used to identify genes related to the progression of various types of cancer. Zheng et al. used bioinformatics to screen molecular markers related to the prognosis of ovarian cancer [36]. Chen et al. found several methods to predict the prognosis of patients with colon cancer through differential gene screening, functional enrichment and prognostic risk score model analysis [37].

In this study, the same differentially expressed genes were identified from 5 GEO datasets (GSE19826, GSE26899, GSE54129, GSE79973, and GSE103236) to explore the potential molecular mechanisms and biomarkers of gastric cancer. After analysis, we identified 37 differentially expressed genes, including 25 upregulated genes and 12 downregulated genes. The results showed that the 37 differentially differentiated genes were mainly enriched with extracellular structure organization, collagen-containing extracellular matrix, endoplasmic reticulum lumen and other biological processes. KEGG pathway analysis showed that these genes were associated with protein digestion and absorption, gastric acid secretion, and collecting duct acid secretion. Four hub genes were identified by PPI network analysis of differentially expressed genes. By analyzing the tissue expression levels, OS, DSS and PFI of hub genes, we found that COL5A2 was highly expressed in cancer tissues, and the high expression of COL5A2 was significantly correlated with poor prognosis. Therefore, COL5A2 was selected as a prognostic marker for gastric cancer.

Studies have shown that the desmoplasia microenvironment in tumors is not a passive scaffold for tumor cells, but rather an active driver of carcinogenesis [38]. The major component of desmoplasia are collagens, which maintain tissue integrity under physiological conditions [39]. COL5 is a kind of collagen formed by regulatory fibrils that only accounts for 2-5% of the total collagen in normal tissues. In the process of fiber formation, COL5 protein and collagen I-type protein form abnormal fibers, which regulate the diameter of collagen I-type fibers [40]. In addition, COL5 affects total collagen content, as shown in COL5 knockout mice, which exhibit an abnormally large fiber count reduction [41]. Collagen activates intracellular signaling pathways that bind to integrins [42]. The major receptor of COL5 is α2β1-integrin [43, 44]. In glomerular endothelial cells, β1-integrin is considered essential for COL5-mediated signal transduction and subsequent downstream activation of focal adhesion kinase and paxillin [45]. A study showed that integrin α2β1 is involved in the metastasis process of human gastric cancer, which is related to lymph node and liver metastasis [46]. Integrin α2β1 can promote peritoneal metastasis of gastric cancer by acting on cysteine-rich 61 [47]. This COL5-mediated activation of the β1-integrin signaling pathway promotes cell migration and movement [48]. Clinical programs targeting integrin α2β1 in metastatic colorectal cancer are already underway [49, 50]. Exploring ECM components, receptors, and associated signaling molecules as biomarkers for prognosis and/or therapeutic targets and treating cancer by using combinations of ECM targeting with RTK inhibitors or immunooncology drugs are promising approaches [51].

*COL5A2* belongs to the collagen family, which is the main component of the extracellular matrix [52]. The expression of COL5A2 is related to the occurrence and development of colon cancer [53]. Fischer et al. confirmed that COL5A2 was expressed in colon cancer samples, but not in normal colon epithelial cells [6]. In recent years, some studies have confirmed the relationship between collagen family genes and gastric cancer. Tan Y et al. showed that COL5A2 knockout reduced the migration ability of gastric cancer cells [54]. Ding YL et al. proved that COL5A2 has a strong correlation with renal metastasis of gastric cancer, and its expression level may be a risk factor for renal metastasis of gastric cancer [55]. Shen H et al. showed that COL5A2 has an important effect on the prognosis of gastric cancer [56]. Through the data analysis of gastric cancer in the TCGA database, we found that the expression of COL5A2 in T1 stage was different from those in the T2–4 stages. In N stage and M stage, the expression of COL5A2 was different between normal people and patients. After that, we performed a single-gene differential analysis of COL5A2 and carried out a follow-up analysis of the differentially expressed genes. Through GO analysis, we found that COL5A2 is closely related to the occurrence and development of the epithelium, the structure of the cytoskeleton and the activity of specific enzymes. Through GSEA, we found that COL5A2 promoted the expression of the integrin signaling pathway, the PI3K-AKT signaling pathway, and the collagen-related pathway. Our findings are consistent with previous studies.

We predicted *COL5A2*-bound miRNAs from the starBase database and identified 151 miRNAs. By analyzing the prognosis and the relationship with COL5A2, we concluded that hsa-miR-200b-3p is the miRNA most likely to affect COL5A2. MiR-200b-3p belongs to the microRNA-200 family, and the elevation of miR-200b-3p inhibits epithelial to mesenchymal transformation, thus inhibiting tumor metastasis [57]. Studies have shown that ZEB1 and SIP1 are key promoters of cancer progression [58, 59]. Gregory PA et al. found that the expression levels of ZEB1 and SIP1 are controlled by the miR-200 family, suggesting that downregulation of these miRNAs is an important early step in tumor metastasis [60]. Combined with the above studies, we speculated that hsa-miR-200b-3p might inhibit tumor progression by binding *COL5A2*.

Some studies have shown that lncRNAs can participate in the regulation of ECM. LncRNA CTD-2589 M5.4 can inhibit the progression of ovarian cancer by regulating ECM remodeling [61]. LINC01089 can be used as an inhibitor of ECM invasion in breast cancer [62]. MiR-150 is involved in ECM-dependent biological processes in hepatocellular carcinoma [63]. A study has constructed a prognostic model for gastric cancer based on maternally related lncRNAs, providing a new perspective for the prognostic judgment of gastric cancer [64]. Because of the importance of lncRNAs, we predicted the pseudogenes/lncRNAs bound to miRNAs and conducted correlation and clinical correlation analyses between the predicted pseudogenes/lncRNAs and miR-200b-3p. We identified 3 pseudogenes (HSPA8P4, RBMS1P1, ZNF652P1) and 7 lncRNAs (AC025569.1, LINC01140, LINC01303, MSC-AS1, OIP5-AS1, RRN3P2, ZEb1-AS1). Subsequently, these genes were verified by Cox analysis and tissue PCR, and the ceRNA regulatory network was established. Finally, we constructed a nomogram to predict the prognosis of gastric cancer.

Our study presents several limitations. First, the amount of data published in public databases is limited. Therefore, the clinical data used for analysis in this study may lead to potential errors or biases. Second, the nomogram data are all from Western countries. Therefore, the results of this study may not apply to patients in Asian countries. Third, the pseudogene/lncRNA-hsa-miR-200b-3p-COL5A2 network needs further experimental verification.

## Conclusion

Through bioinformatics analysis, a new pseudogene/lncRNA-hsa-miR-200b-3p-COL5A2 ceRNA network was established. A nomogram was constructed to predict the survival of patients with gastric cancer. This network may be utilized as promising therapeutic targets and prognostic biomarkers in the future.

## Supplementary Information

**Additional file 1.**

## Data Availability

TCGA and GEO belong to public databases. The patients involved in the database have obtained ethical approval. Users can download relevant data for free for research and publish relevant articles. The GEO dataset name was mentioned in Materials and Methods.

## Abbreviations

GC: Gastric Cancer
ECM: Stromal Extracellular Matrix
COL5: Type V Collagen
COL5A2: Type V Collagen α2 Chain
NcRNAs: Noncoding RNAs
MiRNA: microRNA
LncRNA: long ncRNA
CeRNA: Competing endogenous RNA
PPI: Protein Protein Interaction
GEO database: Gene Expression Omnibus database
DEGs: Differentially Expressed Genes
TCGA database: The Cancer Genome Atlas database
GSEA: Gene Set Enrichment Analysis
OS: Overall Survival
DSS: Disease Specific Survival
PFI: Progression Free Interval
aDC: activated DC
iDC: immature DC
pDC: Plasmacytoid DC
Tcm: T central memory
Tem: T effector memory
Tfh: T follicular helper
Tgd: T gamma delta
GO: Gene Ontology
KEGG: Kyoto Encyclopedia of Gene and Genome
