# Supplementary material for: Comprehensive analysis to identify pseudogenes/lncRNAs-hsa-miR-200b-3p-COL5A2 network as a prognostic biomarker in gastric cancer
Source: Hereditas. 2022 Nov 29;159:43. doi: 10.1186/s41065-022-00257-6 (PMC9706917; doi:10.1186/s41065-022-00257-6)
Supplement: Supplementary file 1 — Additional file 1. [file 41065_2022_257_MOESM1_ESM.docx]

Table S1 Univariate cox regression analysis and multivariate cox regression analysis for patients with gastric cancer

| Characteristics | Total(N) | Univariate analysis | | Multivariate analysis | |
| --- | --- | --- | --- | --- | --- |
|  |  | Hazard ratio (95% CI) | P value | Hazard ratio (95% CI) | P value |
| T stage | 362 |  |  |  |  |
| T1 | 18 | Reference |  |  |  |
| T2 | 78 | 6.725 (0.913-49.524) | 0.061 | 3.214 (0.386-26.720) | 0.280 |
| T3 | 167 | 9.548 (1.326-68.748) | **0.025** | 4.471 (0.468-42.730) | 0.194 |
| T4 | 99 | 9.634 (1.323-70.151) | **0.025** | 3.910 (0.394-38.832) | 0.244 |
| N stage | 352 |  |  |  |  |
| N0 | 107 | Reference |  |  |  |
| N1 | 97 | 1.629 (1.001-2.649) | **0.049** | 2.164 (0.928-5.048) | 0.074 |
| N2 | 74 | 1.655 (0.979-2.797) | 0.060 | 2.107 (0.765-5.807) | 0.149 |
| N3 | 74 | 2.709 (1.669-4.396) | **<0.001** | 3.395 (1.239-9.304) | **0.017** |
| M stage | 352 |  |  |  |  |
| M0 | 327 | Reference |  |  |  |
| M1 | 25 | 2.254 (1.295-3.924) | **0.004** | 0.584 (0.190-1.796) | 0.348 |
| Pathologic stage | 347 |  |  |  |  |
| Stage I | 50 | Reference |  |  |  |
| Stage II | 110 | 1.551 (0.782-3.078) | 0.209 | 0.808 (0.249-2.621) | 0.722 |
| Stage III | 149 | 2.381 (1.256-4.515) | **0.008** | 0.694 (0.144-3.339) | 0.649 |
| Stage IV | 38 | 3.991 (1.944-8.192) | **<0.001** | 1.360 (0.274-6.749) | 0.707 |
| Gender | 370 |  |  |  |  |
| Female | 133 | Reference |  |  |  |
| Male | 237 | 1.267 (0.891-1.804) | 0.188 |  |  |
| Age | 367 |  |  |  |  |
| <=65 | 163 | Reference |  |  |  |
| >65 | 204 | 1.620 (1.154-2.276) | **0.005** | 1.985 (1.276-3.088) | **0.002** |
| Histological type | 369 |  |  |  |  |
| Diffuse Type | 63 | Reference |  |  |  |
| Mucinous Type | 19 | 0.288 (0.087-0.954) | **0.042** | 0.183 (0.040-0.831) | **0.028** |
| Not Otherwise Specified | 202 | 1.179 (0.751-1.852) | 0.475 | 1.197 (0.671-2.134) | 0.542 |
| Papillary Type | 5 | 1.705 (0.514-5.663) | 0.383 | 5.305 (1.351-20.831) | **0.017** |
| Signet Ring Type | 11 | 2.430 (1.093-5.404) | **0.029** | 1.555 (0.580-4.170) | 0.380 |
| Tubular Type | 69 | 0.953 (0.548-1.655) | 0.863 | 0.872 (0.440-1.727) | 0.694 |
| Residual tumor | 325 |  |  |  |  |
| R0 | 294 | Reference |  |  |  |
| R1 | 15 | 1.910 (0.961-3.797) | 0.065 | 1.387 (0.600-3.205) | 0.444 |
| R2 | 16 | 7.866 (4.325-14.304) | **<0.001** | 4.636 (1.756-12.239) | **0.002** |
| Histologic grade | 361 |  |  |  |  |
| G1 | 10 | Reference |  |  |  |
| G2 | 134 | 1.648 (0.400-6.787) | 0.489 |  |  |
| G3 | 217 | 2.174 (0.535-8.832) | 0.278 |  |  |
| Reflux history | 213 |  |  |  |  |
| No | 174 | Reference |  |  |  |
| Yes | 39 | 0.582 (0.291-1.162) | 0.125 |  |  |
| Antireflux treatment | 179 |  |  |  |  |
| No | 142 | Reference |  |  |  |
| Yes | 37 | 0.756 (0.422-1.353) | 0.346 |  |  |
| H pylori infection | 162 |  |  |  |  |
| No | 144 | Reference |  |  |  |
| Yes | 18 | 0.650 (0.279-1.513) | 0.317 |  |  |
| Barretts esophagus | 207 |  |  |  |  |
| No | 192 | Reference |  |  |  |
| Yes | 15 | 0.892 (0.326-2.441) | 0.824 |  |  |
| COL5A2 | 370 |  |  |  |  |
| Low | 186 | Reference |  |  |  |
| High | 184 | 1.537 (1.102-2.143) | **0.011** | 1.257 (0.792-1.994) | 0.332 |
| AC241952 1 | 370 |  |  |  |  |
| Low | 183 | Reference |  |  |  |
| High | 187 | 0.918 (0.661-1.273) | 0.606 |  |  |
| HSPA8P4 | 370 |  |  |  |  |
| Low | 185 | Reference |  |  |  |
| High | 185 | 1.433 (1.030-1.993) | **0.033** | 0.884 (0.578-1.352) | 0.569 |
| PHC1P1 | 370 |  |  |  |  |
| Low | 184 | Reference |  |  |  |
| High | 186 | 1.347 (0.966-1.877) | 0.079 | 1.218 (0.746-1.988) | 0.431 |
| RBMS1P1 | 370 |  |  |  |  |
| Low | 182 | Reference |  |  |  |
| High | 188 | 1.749 (1.251-2.445) | **0.001** | 1.558 (0.968-2.506) | 0.068 |
| AC008040 1 | 370 |  |  |  |  |
| Low | 184 | Reference |  |  |  |
| High | 186 | 1.064 (0.766-1.478) | 0.710 |  |  |
| AC016727 1 | 370 |  |  |  |  |
| Low | 185 | Reference |  |  |  |
| High | 185 | 1.079 (0.778-1.497) | 0.649 |  |  |
| AC025569 1 | 370 |  |  |  |  |
| Low | 184 | Reference |  |  |  |
| High | 186 | 1.212 (0.873-1.682) | 0.251 |  |  |
| AL049796 1 | 370 |  |  |  |  |
| Low | 183 | Reference |  |  |  |
| High | 187 | 1.243 (0.894-1.729) | 0.196 |  |  |
| LINC01140 | 370 |  |  |  |  |
| Low | 185 | Reference |  |  |  |
| High | 185 | 1.295 (0.932-1.800) | 0.123 |  |  |
| LINC01303 | 370 |  |  |  |  |
| Low | 183 | Reference |  |  |  |
| High | 187 | 1.593 (1.143-2.221) | **0.006** | 1.119 (0.716-1.750) | 0.621 |
| MSC-AS1 | 370 |  |  |  |  |
| Low | 185 | Reference |  |  |  |
| High | 185 | 1.805 (1.290-2.526) | **<0.001** | 1.361 (0.808-2.292) | 0.247 |
| OIP5-AS1 | 370 |  |  |  |  |
| Low | 184 | Reference |  |  |  |
| High | 186 | 1.008 (0.726-1.399) | 0.962 |  |  |
| RRN3P2 | 370 |  |  |  |  |
| Low | 182 | Reference |  |  |  |
| High | 188 | 1.273 (0.915-1.771) | 0.152 |  |  |
| ZEB1-AS1 | 370 |  |  |  |  |
| Low | 184 | Reference |  |  |  |
| High | 186 | 1.028 (0.741-1.425) | 0.870 |  |  |
| ZNF652P1 | 370 | 1.319 (0.625-2.782) | 0.467 |  |  |
